# δ^13^C-CH4 reveals CH4 variations over oceans from mid-latitudes to the Arctic

**DOI:** 10.1038/srep13760

**Published:** 2015-09-01

**Authors:** Juan Yu, Zhouqing Xie, Liguang Sun, Hui Kang, Pengzhen He, Guangxi Xing

**Affiliations:** 1 Institute of Polar Environment, School of Earth and Space Sciences, University of Science and Technology of China, Hefei, Anhui, 230026; 2 Institute of Soil Science, Chinese Academy of Sciences, Nanjing, 210008.

## Abstract

The biogeochemical cycles of CH_4_ over oceans are poorly understood, especially over the Arctic Ocean. Here we report atmospheric CH_4_ levels together with δ^13^C-CH_4_ from offshore China (31°N) to the central Arctic Ocean (up to 87°N) from July to September 2012. CH_4_ concentrations and δ^13^C-CH_4_ displayed temporal and spatial variation ranging from 1.65 to 2.63 ppm, and from −50.34% to −44.94% (mean value: −48.55 ± 0.84%), respectively. Changes in CH_4_ with latitude were linked to the decreasing input of enriched δ^13^C and chemical oxidation by both OH and Cl radicals as indicated by variation of δ^13^C. There were complex mixing sources outside and inside the Arctic Ocean. A keeling plot showed the dominant influence by hydrate gas in the Nordic Sea region, while the long range transport of wetland emissions were one of potentially important sources in the central Arctic Ocean. Experiments comparing sunlight and darkness indicate that microbes may also play an important role in regional variations.

Methane (CH_4_) is an important long-lived greenhouse gas^1^, which contributes directly and indirectly to radiative forcing that affects the climate^2^. Methane is also a significant reactive gas that plays an important role in tropospheric and stratospheric chemistry^3^. The oxidation of CH_4_ by hydroxyl radicals (OHs) in the troposphere can lead to the formation of formaldehyde (CH_2_O), ozone (O_3_), carbon monoxide (CO), and water vapour. In conjunction with CO, CH_4_ can control the amount of OH in the troposphere. It also reacts with Cl radicals in the stratosphere, preventing them from reducing O_3_. CH_4_ leves have more than doubled since the industrial revolution and the global average concentration was estimated as 1.808 ppm in 2012^4^. This increase is attributed to an excess of sources, both natural and anthropogenic, compared to sinks. Most parts of the ocean are supersaturated in CH_4_ in relation to its partial pressure in the atmosphere^3^. Oceans cover roughly 70% of the earth’s surface, play a critical role in controlling global temperature, and serve as a source or a sink for many atmospheric trace gases^5^. Because of the isotopic fractionation effect, CH_4_ from different sources have different isotopic characteristics. The most commonly measured isotope of CH_4_ is ^13^C. Depleted-δ^13^C is derived from bacterial sources and enriched-δ^13^C is derived from non-bacterial sources such as natural gas and biomass burning^6^. Isotopic determination of δ^13^C-CH_4_ in the atmosphere, in conjunction with measurements of concentrations, provides a better understanding of CH_4_ sources and sinks.

Ehhalt first determined a budget of sources and sinks of CH_4_ related to the total atmospheric burden^7^. Since then, extensive CH_4_ concentration and δ^13^C-CH_4_ measurements have been performed at sites in the Northern and Southern hemispheres^8^,^9^,^10^. These have provided information on the seasonal cycling of CH_4_ and δ^13^C-CH_4_, sources and sinks, and long-term trends^9^,^11^. However, the observations have been land-based measurements that are subject to local contamination error. Contamination risk is reduced over ocean surfaces. The earliest measurements of atmospheric CH_4_ over the North Atlantic and the Pacific oceans showed a weak decrease beginning at 30°N and extending to 20°S^12^. In contrast, the distribution of CH_4_ determined by shipboard air-grab sampling in the South Atlantic region did not reveal clear latitudinal trends^13^. Another study on atmospheric δ^13^C-CH_4_ measurements investigated the Pacific Ocean and revealed three distinct latitudinal bands of δ^13^C-CH_4_^14^. The latitudinal variations of CH_4_ and δ^13^C-CH_4_ are important for understanding the chemical and dynamic processes that control their distributions. Although there were reports on CH_4_ distributions from 85°N to 67°S , the sources and influencing factors are still poorly understood, especially in the Arctic region^15^. Since 2007, the CH_4_ concentrations had stabilized but increased again^16^,^17^. Systematic observations of CH_4_ and δ^13^C-CH_4_ over oceans remain limited. The present knowledge of atmospheric CH_4_ is insufficient for describing all the variations affected by regional influencing. At high latitudes, methane is supersaturated in the surface waters of the Arctic Ocean^18^,^19^ and European coastal areas^20^. Spatial and temporal observation of CH_4_ is essential to identify and quantify the CH_4_ sources. However, the direct atmospheric CH_4_ concentration data and δ^13^C-CH_4_ measurements are meagre over oceans from the mid- to high latitudes of the North Hemisphere, especially over the Arctic Ocean where the physical and chemical characteristics of the oceans waters have changed in response to climatic warming.

In this study, we describe new shipboard determinations of atmospheric CH_4_ concentrations and δ^13^C-CH_4_ measurements conducted from offshore China to the central Arctic Ocean, covering the latitudes and longitudes of 31.1°N–87.4°N and 22.8°W–90°E–166.4°W, during the 5^th^ Chinese National Arctic Research Expedition (CHINARE 2012). This study is the first to report the temporal and spatial distributions of atmospheric CH_4_ concentrations, combined with δ^13^C-CH_4_ measurements, over an extensive spatial scale. The δ^13^C-CH_4_ measurements revealed factors that affect CH_4_ variation.

## Results

### Trends of atmospheric CH_4_ concentrations and δ^13^C-CH_4_

**Figure 1:**
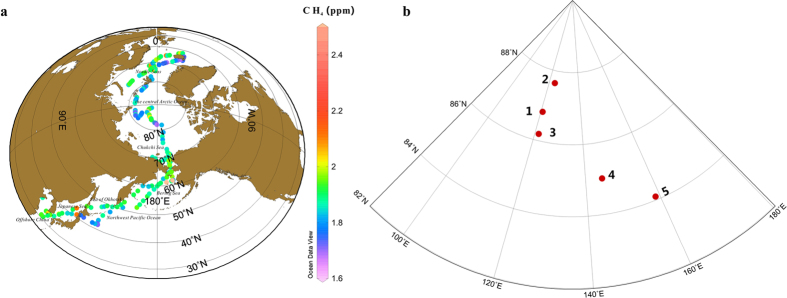
(**a**) Spatial distribution of atmospheric CH_4_ (ppm). (**b**) Experimental sites for CH_4_ flux measurements at the short-term ice stations during CHINARE 2012. Base map is from Ocean Data View (v. 4.0, Reiner Schlitzer. Alfred Wegener Institute for Polar and Marine Research, Bremerhaven, Germany).

**Figure 2:**
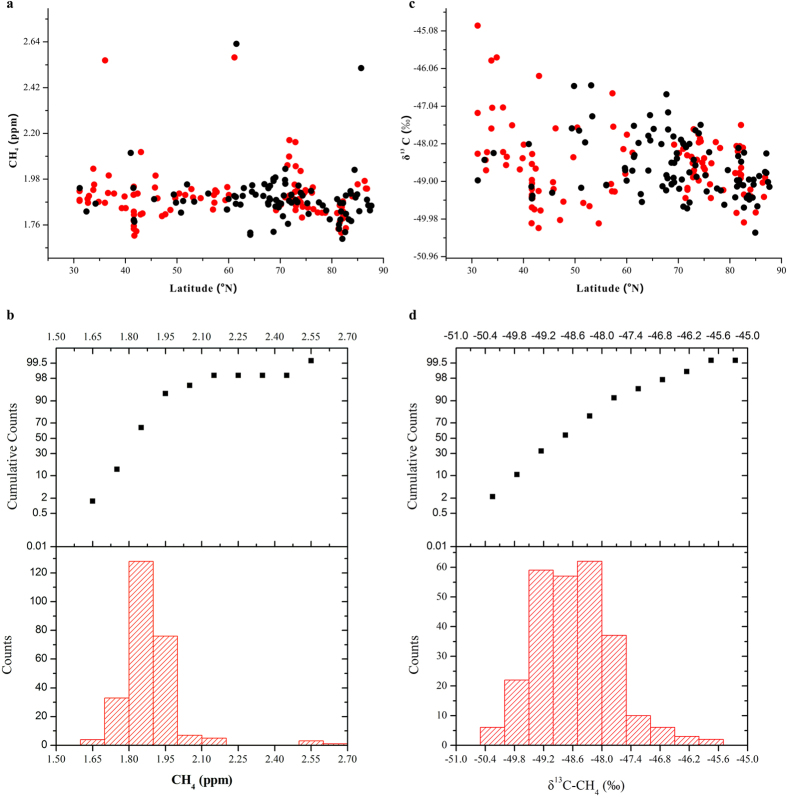
(**a**) Latitudinal distributions of atmospheric CH_4_ during CHINARE 2012; (**b**) The frequency distribution of atmospheric CH_4_; (**c**) Latitudinal distributions of δ^13^C-CH_4_ during CHINARE 2012; (**d**) The frequency distribution of δ^13^C-CH_4_. Red colour and black colour refer to samples collected at day and night, respectively.

The spatial and latitudinal distributions of CH_4_ concentrations determined during CHINARE 2012 are shown in Figs 1a and 2a, respectively. The CH_4_ concentrations varied between 1.65 and 2.63 ppm. By the statistic analysis approximately 79% of the data ranged from 1.80ppm to 2.00ppm, with a median concentration of 1.88 ppm (mean: 1.88 ± 0.12 ppm), indicating that local episode influences were minimal (Fig. 2b). Based on evaluation using the Kolmogorov–Smirnov test, the CH_4_ concentrations were revealed to be distributed inhomogeneously along the cruise track (p < 0.05), even when the four highest values were excluded. The distribution of CH_4_ concentrations showed no obvious relationship with latitude outside the Arctic Ocean, which was consistent with observations from the South Atlantic^13^. However, the CH_4_ concentrations in the Arctic Ocean (>66.5°N) fluctuated in a more consistent manner, especially in the central Arctic Ocean (>80°N), where concentrations increased with latitude (r = 0.44, p < 0.01).

Atmospheric δ^13^C-CH_4_ varied from −50.34% to −44.94% with a median value of −48.63% (meane: −48.55 ± 0.84%) (Fig. 2c). The mean value was lower than the mean δ^13^C-CH_4_ (−47.44%) reported for the northern hemisphere^9^. The higher frequencies ranged from −49.5% to −48% (Fig. 2d). A Kolmogorov–Smirnov test indicated heterogeneous distribution of δ^13^C-CH_4_ along the cruise track (p < 0.05). From mid- to high latitudes, the δ^13^C-CH_4_ measurements showed a slight decreasing trend with latitude (r = −0.23, p < 0.01). A similar decreasing trend with northern latitude was also observed between 65°S and 50°N^9^ during the Pacific Ocean cruise.

### Regional variations of atmospheric CH_4_ concentrations and δ^13^C-CH_4_

Atmospheric CH_4_ and δ^13^C-CH_4_ samples from the entire cruise were separated into eight groups based on geographical locations and ice-coverage characteristics: Offshore China (OC), Japanese Sea (JS), Sea of Okhotsk (SO), Northwest Pacific Ocean (NPO), Bering Sea (BS), Chukchi Sea (CS), central Arctic Ocean (CAO), and Nordic Seas (NS) (Table 1). For the sample regions outside the Arctic Ocean, the mean (±SD) CH_4_ leves in the OC, JS, SO and BS areas were 1.90 ± 0.05 ppm, 2.00 ± 0.21 ppm, 1.90 ± 0.05 ppm and 1.93 ± 0.17 ppm, respectively. By use of non-parametric tests, the BS and JS values were significantly higher than the CH_4_ value of the NPO (1.84 ± 0.07 ppm) (p < 0.05). For the Arctic Ocean, the mean values of the CS, CAO, and NS areas were 1.89 ± 0.05, 1.86 ± 0.13, and 1.87 ± 0.10 ppm, respectively. Although the averages over CS, CAO, and NS were similar, some relatively higher values were found in the CAO, potentially indicating the possible presence of a CH_4_ source.

The mean measurement of δ^13^C-CH_4_ in the OC region was −47.49 ± 1.24%, which was similar to the mean δ^13^C-CH_4_ (−47.44%) for the Northern Hemisphere^8^. The maximum value of δ^13^C-CH_4_ was −44.04%. However, the δ^13^C-CH_4_ values for the other regions (JS: −48.10 ± 0.70%, SO: −48.07 ± 0.92%, NPO: −49.06 ± 0.92%, BS: −48.50 ± 0.85%, CS: −48.78 ± 0.90%, CAO: −48.93 ± 0.59%, and NS: −48.30 ± 0.54%) were all lower than the mean value in the Northern Hemisphere and the closest land-based observation.

### Temporal variations of atmospheric CH_4_ concentrations and δ^13^C-CH_4_

**Figure 3:**
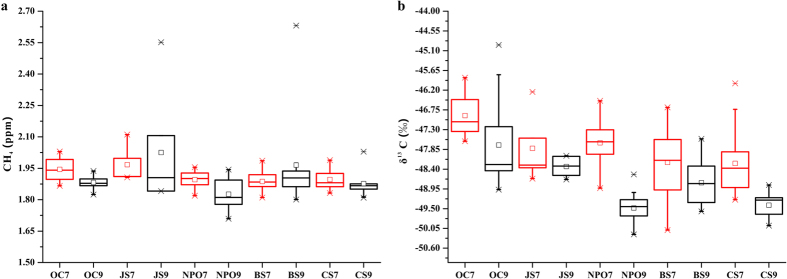
(**a**) Box plots of atmospheric CH_4_ and (**b**) δ^13^C-CH_4_ between July (marked by red colour) and September (marked by black colour) over the OC, JS, NPO, BS, and CS regions (i.e., OC7 and OC9, JS7 and JS9, NPO7 and NPO9, BS7 and BS9, and CS7 and CS9, respectively). The lower and upper boundaries of the boxes represent the 25^th^ and 75^th^ percentiles, respectively. The lines and squares within or outside of the boxes mark the median and mean values, respectively. The upper and lower asterisks signify the maximum and minimum values.

The CH_4_ concentrations in July and September for the same sampling regions are shown in Fig. 3a. Non-parametric tests indicated that the values in July and September were not significantly different. Figure 3b also shows no significant differences between the measurements of δ^13^C-CH_4_ in July and September in the OC, JS, and BS areas. However, the δ^13^C-CH_4_ values in NPO and CS regions were greater in July than in September.

**Figure 4:**
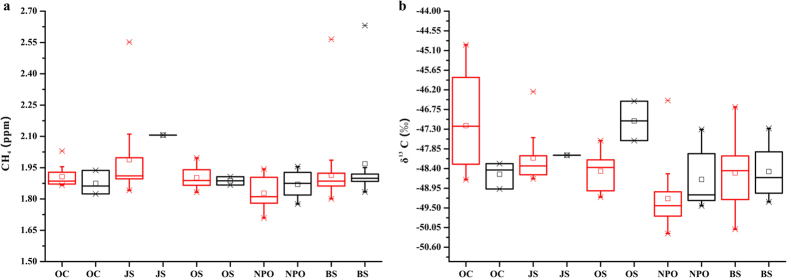
(**a**) Box plots of atmospheric CH_4_ and (**b**) δ^13^C-CH_4_ at day (marked by red colour) and night (marked by black colour) over the OC, JS, NPO, BS, and CS regions. The lower and upper boundaries of the boxes represent the 25^th^ and 75^th^ percentiles, respectively. The lines and squares within or outside of the boxes mark the median and average values, respectively. The upper and lower asterisks signify the maximum and minimum values.

The diurnal and nocturnal CH_4_ concentrations and δ^13^C-CH_4_ measurements for the same sampling regions are shown in Fig. 4. Non-parametric tests revealed no significant differences between day and hight CH_4_ concentrations and δ^13^C-CH_4_ measurements.

## Discussion

Atmospheric CH_4_ concentrations and δ^13^C-CH_4_ over the oceans might be influenced by sources and sinks, e.g., long-range transport of anthropogenic emissions or natural sources emitted from the ocean, and by oxidation by Cl and OH radicals or microbes. Although oceanic CH_4_ was not measured simultaneously in this study, environmental parameters were recorded for further analysis.

### The role of oxidation

**Figure 5:**
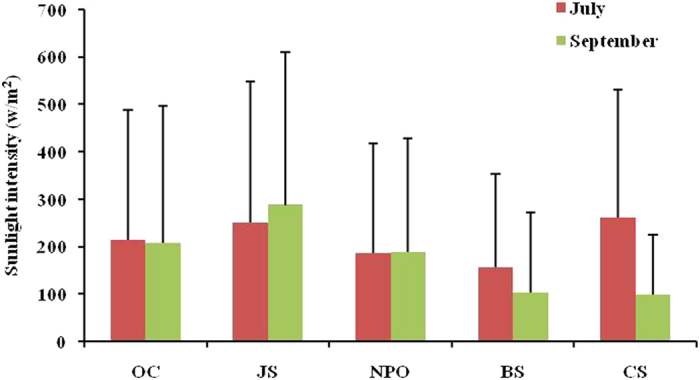
Variations of sunlight intensity in July and September over the OC, JS, NPO, BS, and CS regions. The error bars represent the positive standard deviation.

The phase of δ^13^C-CH_4_ in the seasonal cycle is consistent with the kinetic isotope effect (KIE), which is due to OH and/or Cl radicals oxidizing δ^12^C-CH_4_ faster than δ^13^C-CH_4_, resulting in atmospheric methane enriched in δ^13^C-CH_4_^9^. From mid- to high latitudes, the δ^13^C-CH_4_ showed a slight decreasing trend with latitude in sunlight. The latitudinal loss of δ^13^C-CH_4_ may be due to decreasing enriched input of δ^13^C or chemical oxidation. Considering the potential fuel source influences, the variation of CO with latitude and the air masses of all the samples are shown in Figure S1. The CO concentrations showed a decreasing trend up to about 50°N, indicating a decrease in local contribution by anthropogenic sources, such as fossil fuels, north of 50°N. The back-trajectories of the air masses further confirmed the influence of continental sources of CO in the OC and JS areas (<50°N). However, the latitudinal decreasing trend of δ^13^C-CH_4_ remained under background air, suggesting the potential role of oxidation. It has been reported that OH radicals in the troposphere are the primary sink for global atmospheric CH_4_^21^. Cl radicals may also contribute to CH_4_ loss over oceans. To determine the potential reactive process for CH_4_, the contributions of the OH and Cl radicals were calculated as follows. The average concentrations of the OH and Cl radicals in the marine boundary layer are about 7 × 10^5^–2.9 × 10^6^ molecules·cm^−3^ and 1.8 × 10^4^ molecules·cm^−3^, respectively^22^,^23^,^24^,^25^; mean CH_4_ concentration is 1.88 ppm; and the rate constant at 8 °C based on the mean sampling temperature for OH and Cl radicals is 4.42 × 10^−15^ and 7.59 × 10^−14^ cm^−3^·molecules^−1^·S^−1^, respectively^26^. Assuming the reactive height is 25 m, based on the sampling height, the CH_4_ consumption for OH and Cl radicals is 8.72 × 10^−3^–3.61 × 10^−2^ mg·m^−2^·d^−1^ and 3.85 × 10^−3^ mg·m^−2^·d^−1^, respectively. The effect of Cl radicals on CH_4_ is similar to that of the OH radicals. It is unclear if the level of Cl radicals varies with latitude. However, OH radicals can decrease from low to high latitudes^23^,^27^. In addition, sunlight intensity at the high latitudes is lower than in the mid-latitudes. Thus, reduced oxidation in the high-latitude region might result in depleted δ^13^C-CH_4_. A similar principle could explain higher values of δ^13^C-CH_4_ in July compared to September over the CS area. The significantly higher sunlight intensity over the CS area in July compared with September indicated stronger oxidation potential (Fig. 5). However, the variations between July and September over the OC, JS, BS and NPO regions were not consistent with oxidation results. Most regions outside the Arctic Ocean were influenced by continental sources, and complex sources inputs may influence the oxidation results (Figure S1).

### The role of sources and atmospheric transport

**Figure 6:**
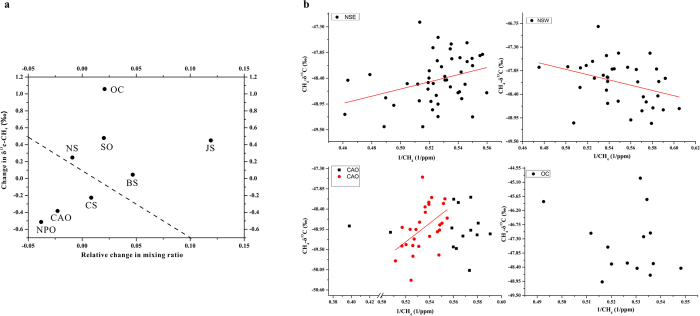
(**a**) Variations of CH_4_ mixing ratio and δ^13^C-CH_4_ in different regions. The dashed line is the KIE line. (**b**) Examples of corresponding Keeling plot over NS, CAO and OC.

To determinthe potential role of sources or sinks, the variations of δ^13^C-CH_4_ versus mixing ratio changes were calculated using the approach proposed by Allen *et al.* (2001)^25^. Assuming that the removal of δ^13^C-CH_4_ is by OH radicals in a closed well-mixed box, the effective rate coefficient for δ^12^C and δ^13^C removal by OH radicals are referred to as k_12_ and k_13_. We adopted the value k_13_/k_12_ = 0.9946^28^, for which ∈ = k_13_/k_12_ − 1 is defined as the KIE. The relationship between changes in δ^13^C-CH_4_ and changes in mixing ratio can be expressed as 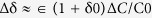, which relates the δ^13^C-CH_4_ variations (Δδ) around the mean δ^13^C-CH_4_ value (δ0) to relative mixing ratio variations (ΔC/C0), where C0 is the mean mixing ratio over the cruise track. If we plot Δδ versus ΔC/C0, the KIE line of slope can be obtained. The details about the expression were presented in a previous report^25^. We can also obtain the variations of δ^13^C-CH_4_ and mixing ratio changes in different regions (Fig. 6a). The values over the OC, JS, SO, NS, and BS areas were above the KIE line (OH oxidation line), representing enriched δ^13^C-CH_4_. Enriched δ^13^C-CH_4_ may mean more oxidation by OH radicals. If the enriched δ^13^C-CH_4_ was influenced by other forms of oxidation such as by Cl radicals, the CH_4_ concentration should be lower. The higher values signified an anthropogenic source, especially for the samples over the OC and JS regions that were far from the KIE line, suggesting that anthropogenic sources might play a major role. The values from the CAO, CS, and NPO areas were below the KIE line and the depleted δ^13^C-CH_4_ might indicate a natural source or less oxidation. If the lower values over the CAO, CS, and NPO areas were a reflection of reduced oxidation only, the concentrations would be higher and thus, the low values signify natural sources.

However, this box model only provided potential sources due to it requiring knowledge of how the out of the box values changing. We therefore applied Keeling plot approach to further investigate the regional variations. If the CH_4_ is emitted into the atmosphere from a single source, the isotopic ratio of the source can be inferred as an end-member for the baseline on the Keeling plot^29^,^30^. It was reported that biogenic sources were dominant at Spitsbergen^29^. Figure 6b shows examples of δ^13^C-CH_4_ plotted against the reciprocal of CH_4_ for the regions over NS and CAO regions, both of them close to Spitsbergen, and the OC region influenced by anthropogenic sources. NS was divided into east NS (NSE) close to Spitsbergen and west NS (NSW) close to Iceland, based on the geographical location. The Keeling plot can be used to understand the processes controlling isotope discrimination and to estimate the isotopic ratio of a source^31^. The CH_4_ collected in NSE gave a source with −52.46% (r = 0.33, p < 0.05), which is similar to the observations at Zeppelin station during Arctic springtime^29^. The main source in the NSE was gas field emissions as few air masses over this were from known emission areas. In contrast, the δ^13^C-CH_4_ of −44.75% (r = 0.35, p < 0.05) signature in NSW indicated enriched δ^13^C inputs. Iceland is a geothermal country. Air masses close to Iceland with heavier δ^13^C may have contributed to the increment in NSW. However, in CAO we collected CH_4_ data in a scale from 1.8 to 2.0 ppm, the highest frequency range. This indicated a source with −62.45% (r = 0.50, p < 0.01) suggesting that the wetlands dominated. The isotope data was consistent with the Siberian railroad and the Ob river with −62.9%^32^. The main air masses in CAO move across the west Siberian coast, also confirming the wetlands emission (Figure S2). But there were some enriched and depleted sources inputs, indicating CAO was influenced by complex mixing sources. It has been demonstrated that extra sources may change seasonal variation^25^. One example outside the Arctic Ocean is the OC region which has complex mixing sources (Fig. 5b). Complex mixing sources may influence the results of seasonal variation outside the Arctic Ocean.

### The role of microbes

**Figure 7:**
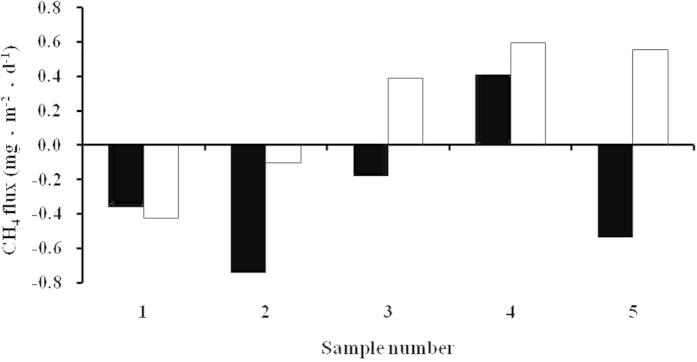
CH_4_ fluxes at short-term ice stations under conditions of sunlight (blank) and darkness (black).

The δ^13^C-CH_4_ should be enriched in sunlight and depleted in darkness in the same region because of the higher photochemical oxidation rate in sunlight. However, we found no significant differences in the CH_4_ concentrations and δ^13^C-CH_4_ measurements between sunlight and darkness in this study. This might be due to microbes producing more CH_4_, which can be deduced based on the case study of CH_4_ variation in sunlight and darkness over the central Arctic Ocean. The CH_4_ fluxes on sea ice in sunlight and darkness are shown in Fig. 7. CH_4_ fluxes on sea ice had positive (emission) or negative (absorption) values^19^. Methanogenic bacteria and methanotrophic bacteria can occur in cold marine waters and in sea ice^33^,^34^. Thus, the CH_4_ emissions might come from the CH_4_ in the water^18^ and from CH_4_ production by microorganisms in the sea ice^35^. During CHINARE 2010, we suggested that negative fluxes could be associated with both photochemical and biochemical oxidation^19^. However, photochemical oxidation cannot explain why lower CH_4_ fluxes were observed in the dark than in sunlight. The negative fluxes could be attributed to the role of methanotrophs. Llight inhibits the growth and activity of methanotrophic bacteria^36^, which could result in the reduced loss of CH_4_ in sunlight compared with darkness. Additionally, temperatures are higher in sunlight than in darkness and methanogenic bacteria can increase CH_4_ production at higher temperature^37^. Archaeal populations of methanogenic and methanotrophic bacteria can be abundant in cold and temperate environments^37^. In temperate environments, the depleted δ^13^C-CH_4_ produced by microbes in the sunlight might offset the sink of chemical oxidation.

## Experimental Methods

### Sampling gas

During CHINARE 2012 (July-September 2012) air samples were collected from the marine boundary layer using 17.5-ml vacuum vials (manufactured by the Institute of Japanese Agricultural Environment) and 0.5-l Tedlar gas bags to determine the CH_4_ concentrations and values of δ^13^C-CH_4_, respectively. The samples in the gas vacuum vials were sealed with a butyl-rubber septum and a plastic cap, following the same sampling method used in the research on Antarctica^38^. The cruise covered the eight geographical areas shown in Fig. 1a. The sampling location was the fifth deck of the icebreaker *Xuelong*, which was about 25 m above sea level. To avoid contamination by ship emissions and anthropogenic factors the samples were collected upwind. The gas vacuum vial was equilibrated in the air for about 1 min using a two-way needle above the head. Samples were collected two or three times each day. The sampling times after 06:00 and 18:00 (local time) were considered as day (sunlight) and night (darkness), respectively. Ancillary data including sunlight intensity and CO concentrations analysed using an EC9830 monitor were also recorded along the cruise track^39^. CH_4_ fluxes on sea ice in sunlight and darkness (simulating day and night) were determined using a static chamber technique less than 2 h at five short-term ice stations (sites shown in Fig. 1b). The procedure was based upon a previous report^14^. The inner diameter of the cylindrical chamber was 0.4 × 0.3 m. The open-bottomed acrylic resin chambers were placed on collars installed at the measurement sites. The use of the collars allowed the same spot to be measured repetitively, ensures that the chamber is well sealed, and minimizes site disturbance. One chamber allowed sunlight transmission and the other did not permit sunlight. Once the chamber was set up, the head-space samples were immediately transferred into the vacuum vial using a two-way needle^19^. The sampling procedures were repeated at 20- or 30-min intervals for about 2 h at each site. All collected samples were analysed in the laboratory of the Institute of Soil Science, Chinese Academy of Sciences, Nanjing, China.

### Determination of CH_4_ concentrations and fluxes

An Agilent 7890 A gas chromatograph (GC) with a flame ionization detector (FID) was used to determine the CH_4_ concentrations. The GC-FID was equipped with an auto-injection system controlled by a computer program and a back-flushed system of 10-port valves. The chromatographic column was a 2-m stainless steel column filled with high-performance Molecular Sieve 13X. The column and detector temperatures were 85 and 250 °C, respectively. The flow rates of N_2_, H_2_, and air were 25, 60, and 380 ml/min, respectively. CH_4_ standard gas at 10ppm was produced by the National Institute of Metrology, China (NIMC). The GC analysis and calibration were according to GB/T 8984–2008 (NIMC). The calibration scale had a range of 0.95~49.8 ppm. The GC instrument was calibrated using CH_4_ standard gas every twelve samples. The variance coefficient (CV) for each measured sample and each time was <1%. CH_4_ fluxes were calculated using the following equation: P(CH_4_) = 
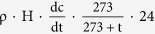
, where P(CH_4_) is the CH_4_ flux (mg·m^−2^·d^−1^), ρ is the density of CH_4_ gas under standard conditions (0.714 kg·m^−3^), H is the height of the chamber (m), dc/dt is the time derivation of CH_4_ in the chamber (ppm·h^−1^), and t is the average temperature (°C )in the chamber^19^,^40^.

### Determination of δ^13^C-CH_4_

The δ^13^C-CH_4_ value was measured using a Thermo Finnigan Mat-253 Isotopic Mass Spectrometer. The Mat-253 mass spectrometer has a fully automated interface for the pre-GC and pre-concentration of trace gases. Full details of the method were described by Cao^41^ and a brief description is given here. In this study, 100-ml gas samples were injected into vacuum glass bottles. If the gas sample was <100 ml, inert gases without CH_4_ was added to the bottle and the bottle was adjusted to normal pressure. The air-sampling bottles were installed into pre-concentration. After their thresholds were blown by He gas, the valves at either end of the sampling bottles were opened and the samples blown into the cold trap by He. At the temperature of −196 °C, only the volatile components (N_2_, O_2_, Ar, and CH_4_) can enter the 1000 °C burning furnace via the cold trap and an aluminous oxidative pipe filled with three 0.13-mm nickel wires. During the test period, CH_4_ is oxidized into CO_2_ and H_2_O. The CO_2_ produced from the CH_4_ combustion was collected by another cold trap and transported into a third cold trap. Then, CO_2_ was passed into the GC for further separation. The calibrated standard CO_2_ was injected into the ionic source three times continuously every 30 s. The ionic flows of m/z 44[^12^C^16^O^16^O]^+^, m/z 45[^13^C^16^O^16^O]^+^, and m/z 46[^12^C^16^O^18^O]^+^ were accepted by cup2, cup3, and cup4, respectively. Adjusting the flow rate of the reference gas controlled the peak intensity of m/z 44 to within 2v–3v. The No. 2 peak was set as the standard sample peak. The CH_4_ peak occurred at about 870 s and the ratio line was positive. According to the ratios of the No. 2 CO_2_ peak and the sample peak, the δ^13^C_PDB_ for the CO_2_ from the CH_4_ was obtained. The 2.02 μl/l compressed CH_4_ was from the same source. Then, 25 ml of compressed CH_4_ was injected into a 100-ml glass bottle with an inert gas filling under normal pressure nine times. The standard deviation for δ^13^C-CH_4_ in the compressed air was 0.196% based on the nine repeated measurements. The different CH_4_ concentrations showed good relationships with ionic flows of m/z 44, and the correlation coefficient was 0.983. GBW04407 (carbon black, national standard substance produced by NIMC with −23.73% for δ^13^C_VPDB_) were used to calibrate the carbon isotope^40^. Isotope ratios were defined as δ^13^C = [(R_sample_/R_standard_)−1] × 1000[%], where δ^13^C is the δ value of the carbon isotope and R is the ratio of the heavy isotope to the light isotope.

## Additional Information

**How to cite this article**: Yu, J. *et al.* δ^13^C-CH_4_ reveals CH_4_ variations over oceans from mid-latitudes to the Arctic. *Sci. Rep.* **5**, 13760; doi: 10.1038/srep13760 (2015).

## Supplementary Material

Supplementary Information

## Figures and Tables

**Table 1:** Summary of atmospheric CH4 and δ^13^C-CH_4_ along the cruise during CHINARE 2012.

**Sampling Area**	**CH**_**4**_ **(ppm)**				**δ**^**13**^**c-CH**_**4**_ **(%)**				
		**Min**	**Max**	**Median**	**Mean ± SD**	**Min**	**Max**	**Median**	**Mean ± SD**
Outside the Arctic Ocean	OC	1.82	2.03	1.89	1.90 ± 0.05	−48.97	−44.94	−47.93	−47.49 ± 1.24
	JS	1.84	2.55	1.91	2.00 ± 0.21	−48.69	−46.25	−48.29	−48.10 ± 0.70
	SO	1.83	2.00	1.89	1.90 ± 0.05	−49.20	−46.52	−48.16	−48.07 ± 0.92
	NPO	1.71	1.95	1.82	1.84 ± 0.07	−50.22	−46.50	−49.37	−49.06 ± 0.92
	BS	1.80	2.63	1.89	1.93 ± 0.17	−50.10	−46.68	−48.49	−48.50 ± 0.85
In the Arctic Ocean	CS	1.81	2.03	1.87	1.89 ± 0.05	−49.97	−46.01	−49.03	−48.78 ± 0.90
	CAO	1.69	2.51	1.85	1.86 ± 0.13	−50.34	−47.53	−48.96	−48.93 ± 0.59
	NS	1.65	2.17	1.87	1.87 ± 0.10	−49.44	−46.82	−48.31	−48.30 ± 0.54
Whole Cruise		1.65	2.63	1.88	1.88 ± 0.12	−50.34	−44.94	−48.63	−48.55 ± 0.84
