# Supplementary material for: δ13C-CH4 reveals CH4 variations over oceans from mid-latitudes to the Arctic
Source: Sci Rep. 2015 Sep 1;5:13760. doi: 10.1038/srep13760 (PMC4555175; doi:10.1038/srep13760)
Supplement: Supplementary Information [file srep13760-s1.doc]

**Supplementary Materials for the manuscript:**

**δ^13^C-CH_4_ reveals CH_4_ variations over oceans from mid-latitudes to the Arctic**

Juan Yu^1^, Zhouqing Xie^1*^, Liguang Sun^1^, Hui Kang^1^, Pengzhen He^1^，Guangxi Xing^2^

1. Institute of Polar Environment, School of Earth and Space Sciences, University of Science and Technology of China, Hefei, Anhui, 230026

2. Institute of Soil Science, Chinese Academy of Sciences, Nanjing, 210008

*Correspondence and requests for materials should be addressed to Z.Q.X. (zqxie@ustc.edu.cn)

To complement the information given in the main manuscript, the following sections provide supporting information Table S1and Figures S1, S2.

**Figure S1** (a) The latitudinal distribution of carbon monoxide (CO) along the cruise and (b) the Seven-day air mass back trajectories of the samples were calculated and converted to GIS line shape files using the TrajStat software (v.1.2.2.6) at starting altitudes above sea level 50m during CHINARE 2012. Base map was also generated by TrajStat software 1.2.2.6 (Wang, Y., Zhang, X. & Draxler, R. R., Environ. Modell. Softw. 24, 938-939, 2009)

**Figure S2** The examples of samples by twelve-day backward trajectories over CAO at three starting altitudes above sea level: 50m, 500m and 1000m, generated by NOAA ARL HYSPLIT trajectory model via NOAA ARL READY Website (http://www.arl.noaa.gov/HYSPLIT.php).

.

**Table S1** Summary of atmospheric CH_4_ and δ^13^C-CH_4_ in different observations close to our sampling sites. The data were obtained from the Earth System Research Laboratory (http://www.esrl.noaa.gov/gmd/dv/data/).

| **year** | **Sampling Area** | **CH_4_ (ppm)** | | | | **δ^13^c-CH_4_ (‰)** | | | |
| --- | --- | --- | --- | --- | --- | --- | --- | --- | --- |
|  |  | **Min** | **Max** | **Median** | **Mean±SD** | **Min** | **Max** | **Median** | **Mean±SD** |
| 1998 | South China Sea | 1.74 | 1.85 | 1.80 | 1.80±0.04 | / | / | / | / |
| 2011 | Tae-ahn Peninsula | 1.90 | 1.96 | 1.92 | 1.93±0.02 | -48.25 | -47.28 | -47.45 | -47.61±0.32 |
| 2012 |  | 1.85 | 1.94 | 1.91 | 1.91±0.03 | / | / | / | / |
| 1996 | Pacific Ocean 45°N | 1.80 | 1.83 | 1.82 | 1.82±0.01 | / | / | / | / |
| 2011 | Barrow | 1.85 | 2.15 | 1.90 | 1.90±0.03 | -47.94 | -47.28 | -47.55 | -47.58±0.23 |
| 2012 |  | 1.88 | 2.50 | 1.91 | 1.91±0.02 | / | / | / | / |
| 2011 | Zeppelin | 1.86 | 1.90 | 1.89 | 1.89±0.01 | -47.84 | -47.27 | -47.43 | -47.50±0.19 |
| 2012 |  | 1.86 | 1.91 | 1.90 | 1.89±0.01 | / | / | / | / |
| 2011 | Alert | 1.85 | 1.90 | 1.89 | 1.88±0.02 | -47.82 | -47.37 | -47.48 | -47.52±0.16 |
| 2012 |  | 1.86 | 1.91 | 1.89 | 1.89±0.02 | / | / | / | / |
| 2012 | Iceland | 1.86 | 1.90 | 1.89 | 1.88±0.01 | / | / | / | / |
| 2004 | Atlantic Ocean | 0.66 | 1.86 | 1.75 | 1.74±0.14 | / | / | / | / |

Note: /. No data


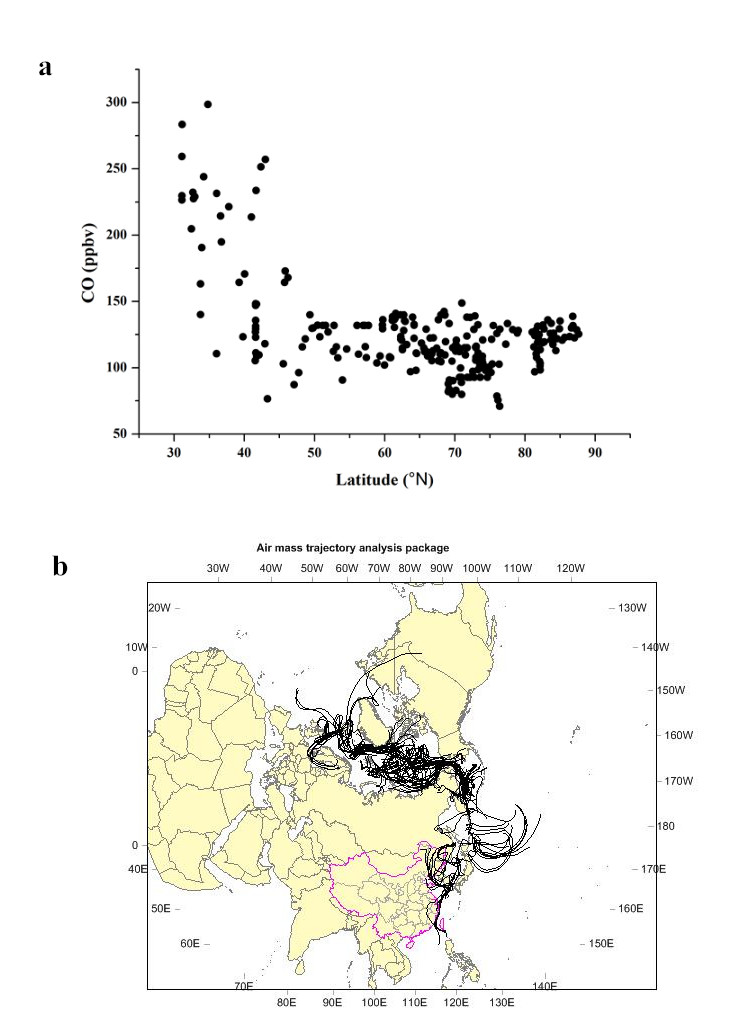


**Figure S1**


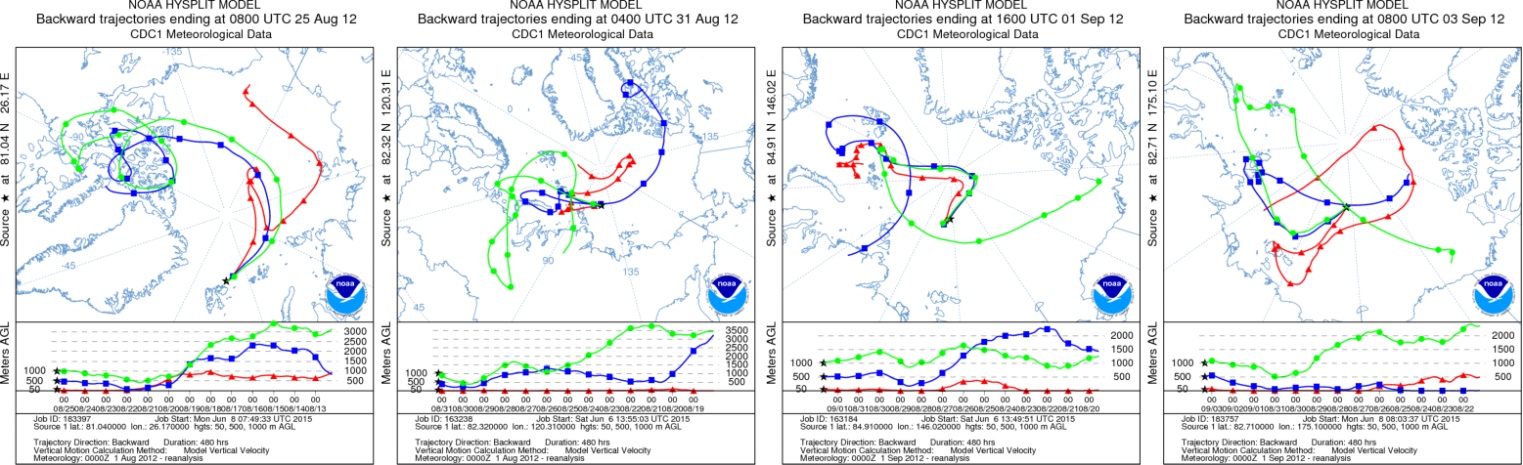


**FigureS2**
